# Molecular Diagnostics, Pathology and Biomarkers of Gastrointestinal Neoplasms

**DOI:** 10.3390/ijms241311136

**Published:** 2023-07-06

**Authors:** Simona Gurzu

**Affiliations:** 1 Department of Pathology, George Emil Palade University of Medicine, Pharmacy, Science and Technology, 38 Gheorghe Marinescu Street, 540139 Targu Mures, Romania; simonagurzu@yahoo.com or simona.gurzu@umfst.ro; 2 Research Center of Oncopathology and Transdisciplinary Research (CCOMT), George Emil Palade University of Medicine, Pharmacy, Science and Technology, 540139 Targu Mures, Romania; 3 Romanian Academy of Medical Sciences, 540139 Targu Mures, Romania

This Special Issue aims to highlight the advances made regarding the molecular profile of digestive system tumors in experimental and clinical studies. We have included data regarding the gastrointestinal tract, liver, and pancreatic tumors.

The most addressed tumor type was colorectal cancer (CRC). Banias et al. [1] performed a historical review of the data regarding the classification of CRC, from Dukes-MAC (modified by Astler and Coller) staging to molecular subgroups, which are used daily to enable survival prediction [1,2]. Although there is a tendency to use genetic data for classification, it is important to note the adaptability of the transdisciplinary team to continually evolve and examine tumors using both imagistic and histopathological methods. Therefore, following a pre-operative imagistic patient evaluation, it would be extremely valuable to use a personalized lymph node station map and mark lymph nodes that may need to be removed by the surgeon and examined by the pathologist [1,3]. These maps are useful for improving the detection accuracy of high lymph node ratios, not only in sporadic cancers but also in patients with synchronous tumors such those developed on the background of gastrointestinal polyposis syndromes [1,4,5].

The molecular classification of CRC can be based on immunohistochemical and molecular biomarkers, such as keratins 7 and 20 and BRAF (v-raf murine sarcoma viral oncogene homolog B1) gene status, which can be used to identify serrated carcinomas [6] or maspin, E-cadherin, ß-catenin, and vimentin, which can be used to assess the epithelial–mesenchymal phenomenon [2]. Markers of microsatellite instability, including immune checkpoint molecules, such as programmed death-ligand 1 (PD-L1) and KRAS (v-Ki-ras2 Kirsten rat sarcoma viral oncogene homolog) gene status, may be helpful to determine the best oncological approach [1,2,4,7].

Several studies have focused on new prognostic or predictive parameters, and new pathways are frequently described for colorectal carcinogenesis. One new biomarker is the spindle apparatus coiled-coil protein 1 (SPDL1). Klimaszewska-Wisniewska et al. proved that SPDL1 upregulation in CRC cells indicates chromosomal instability and long survival [8]. Yasudome et al. introduced a new concept referring to the possible interaction between microRNA139 (miR-139-3p) and keratin 80, which plays a potential tumor-suppressive role [9]. Pietras et al. proved that CRC cell proliferation and vascular migration are mediated by major vault proteins, which possess barrel-like structures [10]. However, their interaction with classic prognostic and predictive factors is not yet understood.

All the above-mentioned biomarkers appear to possess prognostic and predictive value for CRC. In addition to 5-Fluorouracil, cetuximab, bevacizumab, and other molecular-targeted drugs used in oncologic regimens for CRC, it has been suggested that natural flavonoids, such as the propolis phytoestrogen known as chrysin, might have anticancer properties by inducing G2/M phase arrest of tumor cells [11].

Regarding pancreatic cancer, Lim et al. [11] proposed that chemotherapeutics combined with natural products, such as chrysin, which has demonstrated anticancer, anti-inflammatory, and antioxidant properties, may prolong patient survival. The mechanism of chrysin in pancreatic cancer is like that observed in breast cancer, whereby chrysin induces upregulation of the G-protein-coupled estrogen receptor and enhances the inhibitory roles of 17ß-estradiol, ROCK1 (Rho Associated Coiled-Coil Containing Protein Kinase 1), TAGLN2 (Transgelin 2), and FCHO2 (F-BAR domain only protein 1) genes against tumor cells, particularly in women [11]. Clinical trials with BReast CAncer (BRCA) tumor suppressors have demonstrated efficacy against breast and pancreatic cancer. Moreover, some proteins such as mammalian Ena (Mena) have been proved to play a role in pancreatic but also breast carcinogenesis [12]. Therefore, these common mechanisms, highlighted by Lim et al., might be a plausible treatment target for both pancreatic and breast cancer.

One of the newest molecular approaches for hepatocellular carcinoma (HCC) involves damaging tumor cells through the opposite mechanism of hepatocarcinogenesis. Based on this approach, Huang et al. [13] examined the association of HCC development with high-fat diets and fatty liver disease. Using mice, they demonstrated that microRNA-29a (miR-29a), which plays essential roles in liver fibrosis and hepatocarcinogenesis, is downregulated in HCC compared with steatosis. This induces the upregulation of pro-angiogenic factors, such as hypoxia-inducible factor-1 alpha (HIF-1α), vascular endothelial growth factor (VEGFA), angiopoietin 2 (ANGPT2), Lysyl Oxidase (Lox), and Loxl2. This interaction between miR-29a and HIF-1/ANGPT2 is a novel molecular axis that has been proposed as a pathway for HCC development [13]. As HCC is one of the most vascular solid tumors for which classic antiangiogenic therapy does not confer significant benefits [14], inhibiting its development through a new pathway might represent a future therapeutic target for patients with HCC.

Therapeutic challenges are also associated with gastric cancer. Although several studies have focused on its genetic profile, the only suitable gene target for inhibition is *HER-2.* Several patients with metastatic gastric carcinomas with *HER-2* gene amplification have benefited from trastuzumab therapy. However, the amplification rate is low, and HER-2 heterogeneity increases the risk of therapy resistance [15]. Based on previous literature data, Mansorunov et al. [16] proposed a more personalized therapy for patients with gastric cancer based on the simultaneous inhibition of ten immune checkpoints genes. As gastric cancer cells are highly heterogeneous, the next generation of immunotherapy should focus on cancer immunotherapy by synchronously inhibiting pro-metastatic genes, such as galectin-3, galectin-9, and indoleamine 2,3-dioxygenase, as well as their related immune checkpoint co-expressors [16]. Koustas et al. highlighted the need for an improved understanding of the immune system and its molecular drivers in cancer to design optimal immunotherapy strategies [17].

Sarantis et al. [18] investigated the immune microenvironment and immunotherapeutic management of digestive cancers. They proposed assessing the role of viruses in the development of these cancers, as viruses are present in 12% of all malignancies. They hypothesized that optimal immunotherapy should be multimodal and inhibit tumor cell proliferation and the immune microenvironment. To enable this, immune checkpoint inhibitors should be combined with vaccines against immunogens and adoptive cell therapy [18]. Chung et al. proposed the simultaneous therapeutic inhibition of PD-L1 and its triggers based on the interaction of PD-L1 with genes that regulate the tumor microenvironment, such as Chemokine (C-X-C motif) ligand 9 (CXCL9) [7].

The molecular pathways that induce the development and progression of digestive malignancies are complex and require further elucidation. Molecular parameters are useful for enabling better stratification of cases by offering both prognostic and predictive value. However, further studies are needed to understand how to therapeutically inhibit digestive carcinogenesis independently based on tumor location.
